# Complete genome sequence of *Arcobacter* sp. strain 15-2, a novel isolated bacterium from abalone *Haliotis gigantea*

**DOI:** 10.1128/mra.01185-25

**Published:** 2025-12-29

**Authors:** Shumpei Ito, Yukino Mizutani, Reiji Tanaka

**Affiliations:** 1 Graduate School of Bioresource, Mie University 12946, Tsu, Mie, Japan; Montana State University, Bozeman, Montana, USA

**Keywords:** *Arcobacter*, marine microbiology, abalone

## Abstract

Genome sequence of a novel bacterium, *Arcobacter* sp. strain 15-2, isolated from tissue homogenate of abalone *Haliotis gigantea*. The genome is 2,351,109 bp with an estimated 2,273 coding sequences. G + C content is 29.4%.

## ANNOUNCEMENT

Several novel *Arcobacter* species have recently been isolated from marine invertebrates, including bivalves and abalone (1–3). Although *Arcobacter* species are frequently detected in and isolated from bivalves, their pathogenic potential and clinical significance remain poorly understood, especially in relation to abalone, which are economically and ecologically important marine resources. To address this knowledge gap and provide a genomic foundation for future studies on host-pathogen interactions, we report here the complete genome sequence of *Arcobacter* sp. strain 15-2, isolated from the abalone *Haliotis gigantea* in September 2017.

Tissue homogenate of abalone *H. gigantea*, collected at the coastal area of Toba, Mie, Japan (34°30'00"N; 136°50'07"E), and 10-fold dilutions were cultured on Marine agar 2216 (BD) plates at 15°C for 5 days. Several colonies were isolated, and their 16S rRNA gene sequences were analyzed. Among them, a strain designated 15-2 was identified as belonging to the genus *Arcobacter*. Strain 15-2 stored at −80°C was inoculated into Marine broth 2216 (BD) and shaken at 25°C, 100 rpm for 24 h. The genome of strain 15-2 was extracted using Wizard genomic DNA purification system (Promega) according to the manufacturer’s instructions. The concentration of the DNA solution was measured using the QuantiFluor dsDNA System (Promega). The quality and integrity of extracted genomic DNA were assessed using a 5200 Fragment Analyzer (Agilent). For PacBio sequencing, samples were sheared to 10 kb using g-TUBEs (Covaris), and size selection was performed using DNA Clean Beads (MGI Tech) according to the manufacturer’s protocol. The DNA library was prepared using SMRTbell gDNA Sample Amplification Kit (PacBio) and the SMRTbell Express Template Prep Kit 2.0 (PacBio) according to the Procedure & Checklist instructions (part number: 101-730-400 version 06). Polymerase complexes were prepared by Revio Polymerase Kit (PacBio), and sequencing was performed using Revio (PacBio). SMRT Link (version 12.0.0.177059) was used for primary analysis, including Circular Consensus Sequencing to generate high-fidelity reads with a minimum of three passes, adapter removal, and quality filtering to remove <20 per read. Filtlong (version 0.2.1) was used to remove reads shorter than 1,000 bases, resulting in 33,874 reads with an N50 value of 7,338 bp and 105× coverage. *De novo* assembly was performed using Flye (version 2.9.2-b1786) (4, 5) producing a single contig with a total length of 2,351,109 bp and a G + C content of 29.4%. The quality of the assembled genome was assessed using Bandage (version 0.8.1) (6) and CheckM2 (version 1.0.1) (7), without rotating the sequence to start at *dnaA*. Unless otherwise specified, all software was run with default parameters.

Genome annotation with DFAST (version 1.2.0) (8) identified 2,273 coding sequences, 48 tRNA genes, and 16 rRNA genes. NCBI-blastn of its 16S rRNA gene showed 93.5% similarity to *Arcobacter acticola* strain KCTC 52212^T^ (CP042652.1). The low 16S rRNA gene similarity suggests that strain 15-2 represents a novel *Arcobacter* species. As a result, only one pathogenic gene similar to the sequence of a *tly A* was determined (Table 1), but this gene was less similar to other known *Arcobacter* spp. genes.

**TABLE 1:** Pathogenic genes identified in the genomes of representative *Arcobacter* spp. and strain 15-2

Gene	*A. lekithochrous* LFT 1.7	*A. nitrofigilis* DSM 7299	*A. bivalviorum* LMG 26154	*A. halophilus* F166-45	*A. defluvii* CECT7697	15-2
*cadF*	−	−	+	+	−	−
*cjl349*	−	−	−	−	−	−
*ciaB*	+	+	+	+	+	−
*pldA*	−	−	+	+	−	−
*tlyA*	−	+	+	+	−	+
*irgA*	−	−	−	−	−	−
*hecA*	−	+	−	−	+	−
*hecB*	−	+	−	−	−	−
*iroE*	−	−	−	−	+	−

## Data Availability

The whole genome sequence has been deposited in the DDBJ/EMBL/GenBank under the accession number AP038804. The version described in this paper is version 1.00. The BioProject number is PRJDB19036. The BioSample number is SAMD00828114 (*Arcobacter* sp. 15-2). Sequence Read Archive number is DRR612764.
